# Supplementary material for: SHIP164 is a chorein motif lipid transfer protein that controls endosome–Golgi membrane traffic
Source: J Cell Biol. 2022 May 2;221(6):e202111018. doi: 10.1083/jcb.202111018 (PMC9067936; doi:10.1083/jcb.202111018)
Supplement: Table S1 — lists antibodies used in this study. [file JCB_202111018_TableS1.docx]

**Table S1.** List of antibodies used in this study.

| Protein (epitope) | Company; Catalog number | Antibody species | Working dilution for immunocytochemistry | Working dilution for immunoblotting |
| --- | --- | --- | --- | --- |
| GM130 | BD Bioscience; 610822 | Mouse | 1:100 | N/A |
| GM130 | Abcam; ab30637 | Rabbit | 1:300 | N/A |
| EEA1 | BD Bioscience; 610457 | Mouse | 1:100 | N/A |
| EEA1 | Thermo Scientific; PA1-063A | Rabbit | 1:400 | N/A |
| Sortilin | Abcam; ab243043 | Rabbit | 1:500 | N/A |
| MPR (IGF-IIR) | Santa Cruz Biotechnology; sc-53146 | Mouse | 1:100 | N/A |
| TGN46 | Abcam; ab50595 | Rabbit | 1:200 | N/A |
| TGN46 | AbD Serotec; AHP500GT | Sheep | 1:300 | N/A |
| mNeonGreen | ChromoTek;  32f6-100 | Mouse | 1:500 | 1:1000 |
| SHIP164 | Custom antibody produced by Proteintech | Rabbit | N/A | 1:500 |
| GAPDH | Proteus; 40-1246 | Mouse | N/A | 1:1000 |
| WIPI2 | Millipore-Sigma; MABC91 | Mouse | N/A | 1:500 |
| α-Tubulin | Sigma Aldrich; T5168 | Mouse | N/A | 1:10000 |
| α-ATG9A | Abcam;  ab108338 | Rabbit | 1:500 | N/A |
| α-LC3B | Cell Signaling;  2775S | Rabbit | 1:500 | N/A |
