# Supplementary material for: SHIP164 is a chorein motif lipid transfer protein that controls endosome–Golgi membrane traffic
Source: J Cell Biol. 2022 May 2;221(6):e202111018. doi: 10.1083/jcb.202111018 (PMC9067936; doi:10.1083/jcb.202111018)
Supplement: Table S2 — lists ORFs and primers used for cloning in this study. [file JCB_202111018_TableS2.docx]

| Supplementary Table 2 |  |  |  |  |  |  |  |  |  |
| --- | --- | --- | --- | --- | --- | --- | --- | --- | --- |
| Protein ORF | Plasmid backbone | Tag for visualization or biochemistry | Template | Cloning Method | Cut sites used linearize plasmid | Forward primer (or ssOligo) | Reverse Primer | Internal primers; Set 1 | Internal primers; Set 2 |
|  |  |  |  |  |  | **Mammalian expression constructs** |  |  |  |
| SHIP164 | pCMV10 | N-terminal 3xFLAG | puc57 SHIP164 | Gibson Assembly | NotI/BamHI | gcggccgccATGGCCGGCATC | ggatccTTACTCAACGGTCATC |  |  |
| SHIP164(Δ901–1099) | pCMV10 | N-terminal 3xFLAG | puc57 SHIP164 | Gibson Assembly | NotI/BamHI | gcggccgccATGGCCGGCATC | ggatccTTACTCAACGGTCATC | cctgccgaccAGCTATAAGAACATGAAACG | tcttatagctGGTCGGCAGGTAATCTGG |
| MBP-SHIP164 | pCMV10 | N-terminal 3xFLAG | puc57 SHIP164; pET28-MBP-TEV | Gibson Assembly; site-directed mutagenesis |  | gatgatgccggcggccgcagtctg | cagactgcggccgccggcatcatc |  |  |
| SHIP164(Δ901–1099)-PH(11-140) | pCMV10 | N-terminal 3xFLAG, C-terminal 6xHis | puc57 SHIP164; pCMV6 VPS13(tethered) | Gibson Assembly |  | GGCCGCGATGGCCGGCATCATCAAAAAAC | GGCAATCTTCTCAACGGTCATCTTCTTAATGTGGT | CCGTTGAGAAGATTGCCCTGCGGGT | CCGGCCATCGCGGCCGCAAG |
| SHIP164 | pcDNA3.1 | Internal mScarlet-i (after residue 915) | pUC57 SHIP164; pmScarlet-C1 | HiFi Assembly | HindIII/XhoI | GCTGGCTAGCGTTTAAACTTAAGCTTACCATGGCCGGCATCATCAAAAAACAAATCC | GTTTAAACGGGCCCTCTAGACTCGAGTTACTCAACGGTCATCTTCTTAATGTGG | CGTAAACAGATTAGCCGCATGGTGAGCAAGGGCGAG; CTCGCCCTTGCTCACCATGCGGCTAATCTGTTTACG | GCGGCATGGACGAGCTGTACAAGGATATCAACCGTATTCGCAGCGTGAC; GTCACGCTGCGAATACGGTTGATATCCTTGTACAGCTCGTCCATGCCGC |
| SHIP164 | pVMart | No tag; IRES RFP | pUC57 SHIP164 | HiFi Assembly | BamHI/XhoI | gcttGGTACCGAGCTCGGatccATGGCCGGCATCATCAAAAAAC | GTTTAAACATCGATGGCCTCGAGTTACTCAACGGTCATCTTC |  |  |
| SHIP164 | pHalo-N1 | C-terminal Halo | pUC57 SHIP164 | HiFi Assembly | HindIII | CAGATCTCGAGACCGGTCGCCACCATGGCCGGCATCATCAAAAAAC | GTACCGTCGACTGCAGAATTCGacctccagatccaccCTCAACGGTCATCTTCTTA |  |  |
| SHIP164 | pmCherry-N1 | C-terminal mCherry | pUC57 SHIP164 | HiFi Assembly | XhoI/EcoRI | GCGCTACCGGACTCAGATCTCGAGACCGGTCGCCACCATGGCCGGCATCATCAAAAAACA | CGGTACCGTCGACTGCAGAATTCGacctccagatccaccCTCAACGGTCATCTTCTTA |  |  |
| SHIP164 Y772A, Y773A | pmCherry-N1 | C-terminal mCherry | pUC57 SHIP164 | HiFi Assembly | XhoI/EcoRI | GCGCTACCGGACTCAGATCTCGAGACCGGTCGCCACCATGGCCGGCATCATCAAAAAACA | CGGTACCGTCGACTGCAGAATTCGacctccagatccaccCTCAACGGTCATCTTCTTA | CAAGAAACTGCTGAAAGAGGCTGCAAGCACCGAGAGCGAACC; GGTTCGCTCTCGGTGCTTGCAGCCTCTTTCAGCAGTTTCTTG |  |
| SHIP164 | pEGFP-C1 | N-terminal EGFP | pUC57 SHIP164 | HiFi Assembly | XhoI/BamHI | GTACAAGTCCGGACTCAGATCTCGAGgcATGGCCGGCATCATCAAAAAAC | CAGTTATCTAGATCCGGTGGATCCATTACTCAACGGTCATCTTCTTAATG |  |  |
| Stx6 | pmCherry-C1 | N-terminal mCherry | cDNA library | HiFi Assembly | EcoRI/BamHI | GATCTCGAGCTCAAGCTTCGAATTCcatgtccatggaggacccc | CAGTTATCTAGATCCGGTGGATCCttacagcactaggaagaggatg |  |  |
| Stx6 1-234 | pmCherry-C1 | N-terminal mCherry | pmCherry-C1 Stx6 | HiFi Assembly | EcoRI/BamHI | GATCTCGAGCTCAAGCTTCGAATTCcatgtccatggaggacccc | GTTATCTAGATCCGGTGGATCCttattggcgccgatcactggt |  |  |
| Stx6 | pTK-mNeonGreen | N-terminal mNeonGreen | pmCherry-C1 Stx6 | Restriction enzyme | EcoRI/BamHI |  |  |  |  |
| Stx6 | pEGFP-C1 | N-terminal EGFP | pmCherry-C1 Stx6 | Restriction enzyme | EcoRI/BamHI |  |  |  |  |
| Rab45 | pcDNA3.1 | N-terminal EGFP | Synthesized | Synthesized |  |  |  |  |  |
| Rab45 S555N | pEGFP-C1 | N-terminal EGFP | pcDNA3.1 Rab45 | HiFi Assembly | KpnI/EcoRI | GACGAGCTGTACAAGGGTACCggtggatctggaggtATGGAGGCTGACGGCGACGGCG | TGCTGGATATCTGCAGAATTCTCAGCCGTTGCAACAGTTCTTC | GCCGCTGTGGGCAAGAATAGCTTCCTCATGAG; CTCATGAGGAAGCTATTCTTGCCCACAGCGGC |  |
| Rab45 | pmCherry-C1 | N-terminal mCherry | pcDNA3.1 Rab45 | HiFi Assembly | BsrGI/EcoRI | GACGAGCTGTACAAGGGTACCggtggatctggaggtATGGAGGCTGACGGCGACGGCG | GTACCGTCGACTGCAGAATTCTCAGCCGTTGCAACAGTTCTTC |  |  |
| FAM174a | pmCherry-N1 | N-terminal mCherry | codon optimized gBlock | HiFi Assembly | EcoRI/BamHI |  |  |  |  |
|  |  |  |  |  |  | **gRNA cloning plasmids** |  |  |  |
| SHIP164 KO gRNA-AB | PX458 | No tag | Synthetic oligo | HiFi Assembly | BbsI | Atcttgtggaaaggacgaaacaccgtttgtaataaagcgtccattgttttagagctagaaatagcaagtt |  |  |  |
| SHIP164 KI IDR5 | pORANGE | No tag | Synthetic oligo | HiFi Assembly | BbsI | atcttgtggaaaggacgaaacaccgGGAACGTTGCCCACCCAACCgttttagagctagaaatagcaagtt |  |  |  |
| IGFIIR (MPR) KI | pORANGE | No tag | Synthetic oligo | HiFi Assembly | BbsI | atcttgtggaaaggacgaaacaccgCTCGTCGCTGTCGTCATGGAgttttagagctagaaatagcaagtt |  |  |  |
|  |  |  |  |  |  | **Complete knock-in plasmids generated from gRNA cloning plasmids** |  |  |  |
| SHIP164 KI IDR5-mNeonGreen | pORANGE | mNeonGreen | pTK-mNeonGreen | Restriction enzyme | HindIII/XhoI | ATAAAGCTTCCAGGTTGGGTGGGCAACGTTCCGGTTCTGGATCGGGTgtgagcaagggcgaggag | ATACTCGAGGGAACGTTGCCCACCCAACCTGGTGCTACCTGAACCcttgtacagctcgtccatg |  |  |
| IGFIIR (MPR) KI mNeonGreen | pORANGE | mNeonGreen | pTK-mNeonGreen | Restriction enzyme | HindIII/XhoI | ATAAAGCTTCTCGTCGCTGTCGTCATGGAGGGggggttcgggggtgagcaagggcgaggagga | ATACTCGAGCCCTCCATGACGACAGCGACGAGcccgaacccttgtacagctcgtccatgccca |  |  |
| IGFIIR (MPR) KI mScarlet-i | pORANGE | mScarlet-i | pmScarlet-C1 | Restriction enzyme | HindIII/XhoI | ATAAAGCTTCTCGTCGCTGTCGTCATGGAGGGggggttcggggGTGAGCAAGGGCGAGGCAG | ATACTCGAGCCCTCCATGACGACAGCGACGAGcccgaaccCTTGTACAGCTCGTCCATGC |  |  |
