# Supplementary figures and images for: SHIP164 is a chorein motif lipid transfer protein that controls endosome–Golgi membrane traffic
Source: J Cell Biol. 2022 May 2;221(6):e202111018. doi: 10.1083/jcb.202111018 (PMC9067936; doi:10.1083/jcb.202111018)

Figure 1c

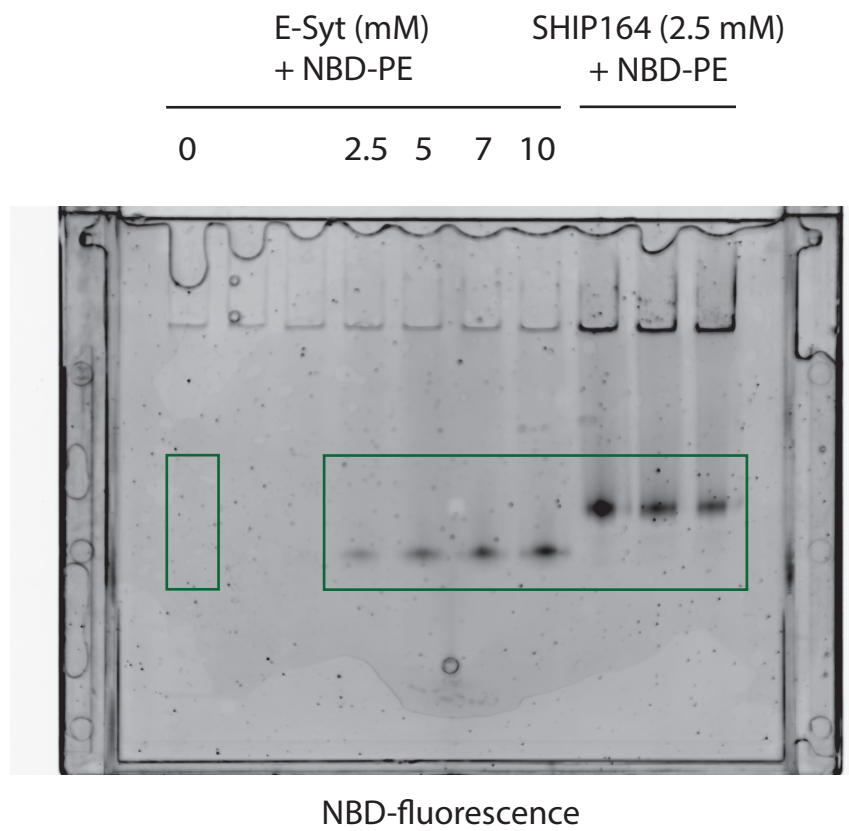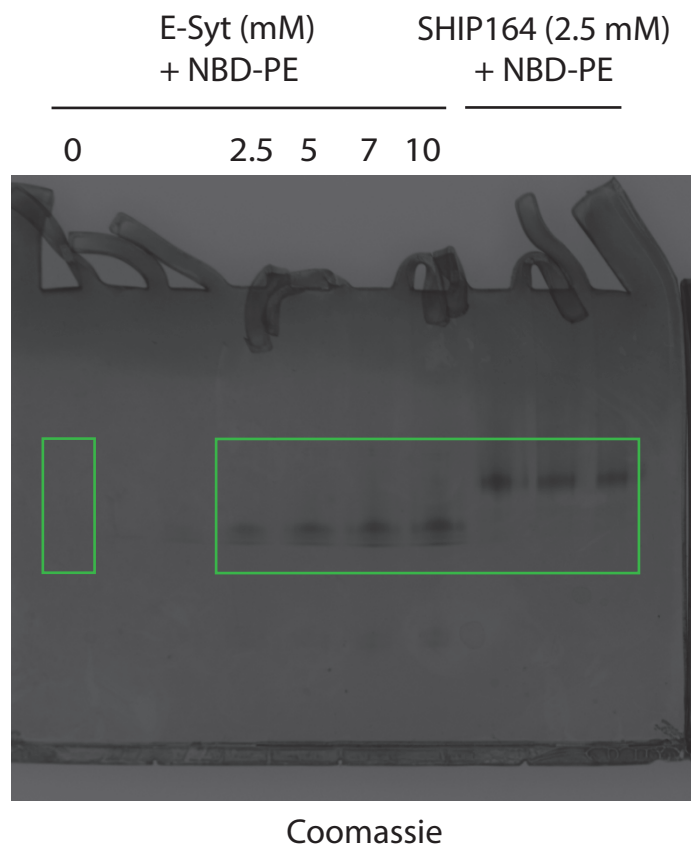

Supplement: SourceData F1 — contains original blots for Fig. 1. [file JCB_202111018_SourceDataF1.pdf]

Source Data: Figure 4B

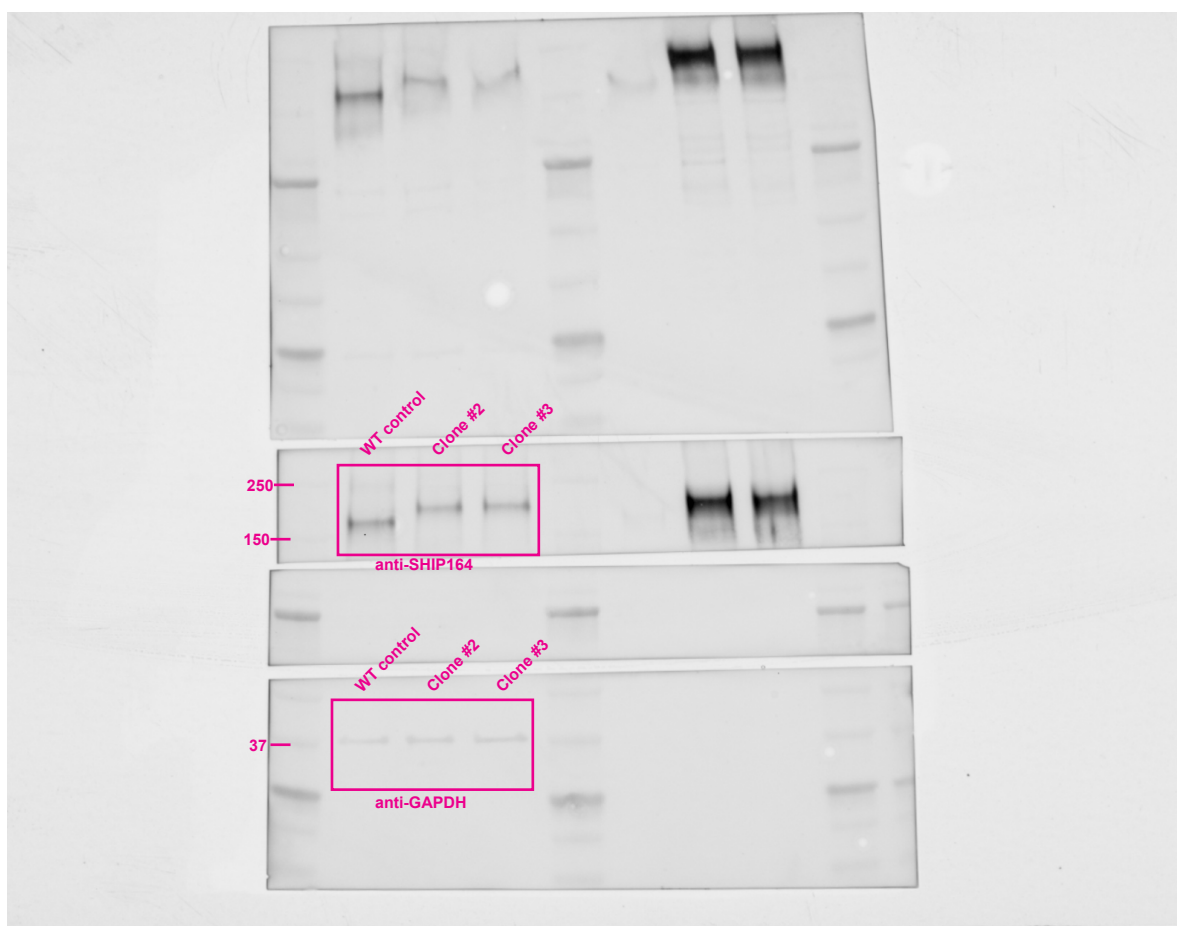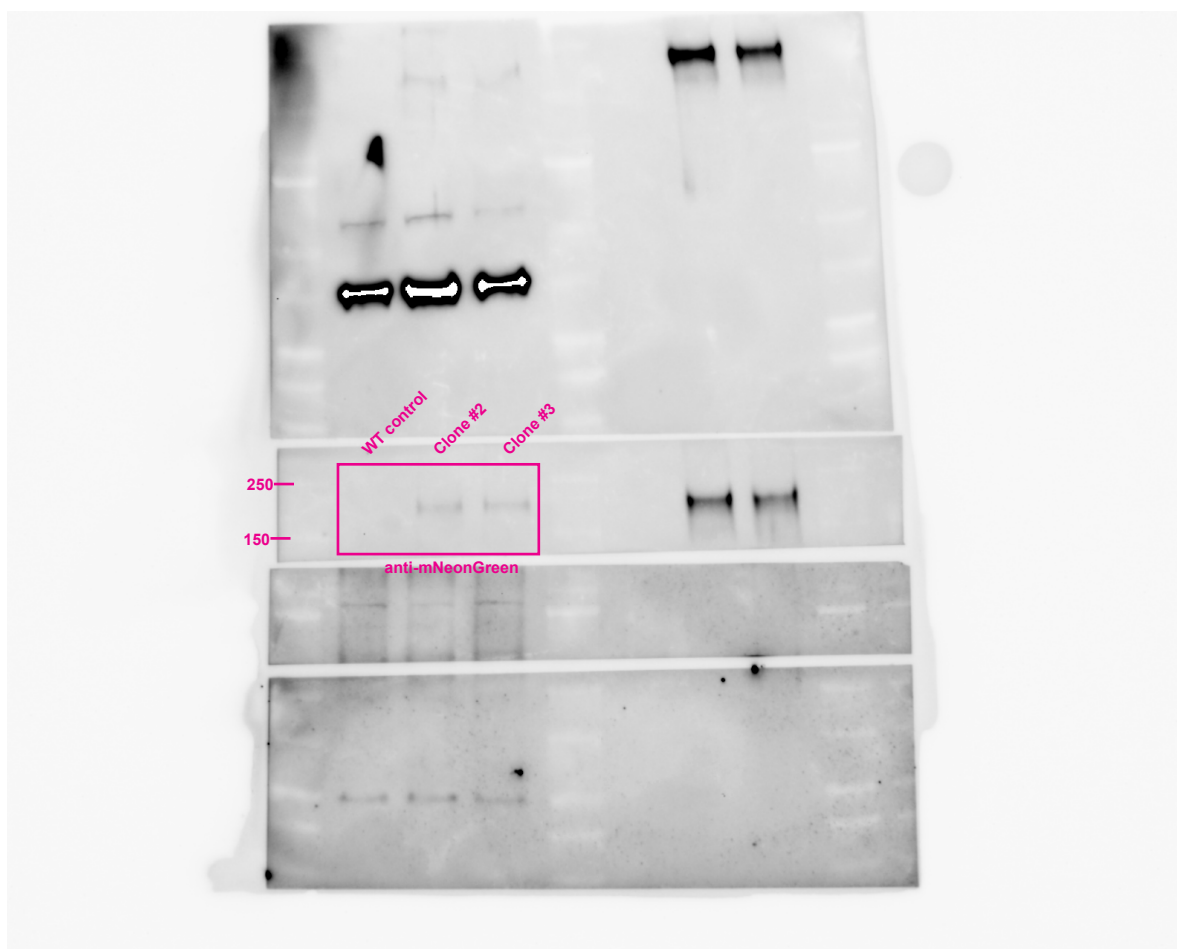

Supplement: SourceData F4 — contains original blots for Fig. 4. [file JCB_202111018_SourceDataF4.pdf]

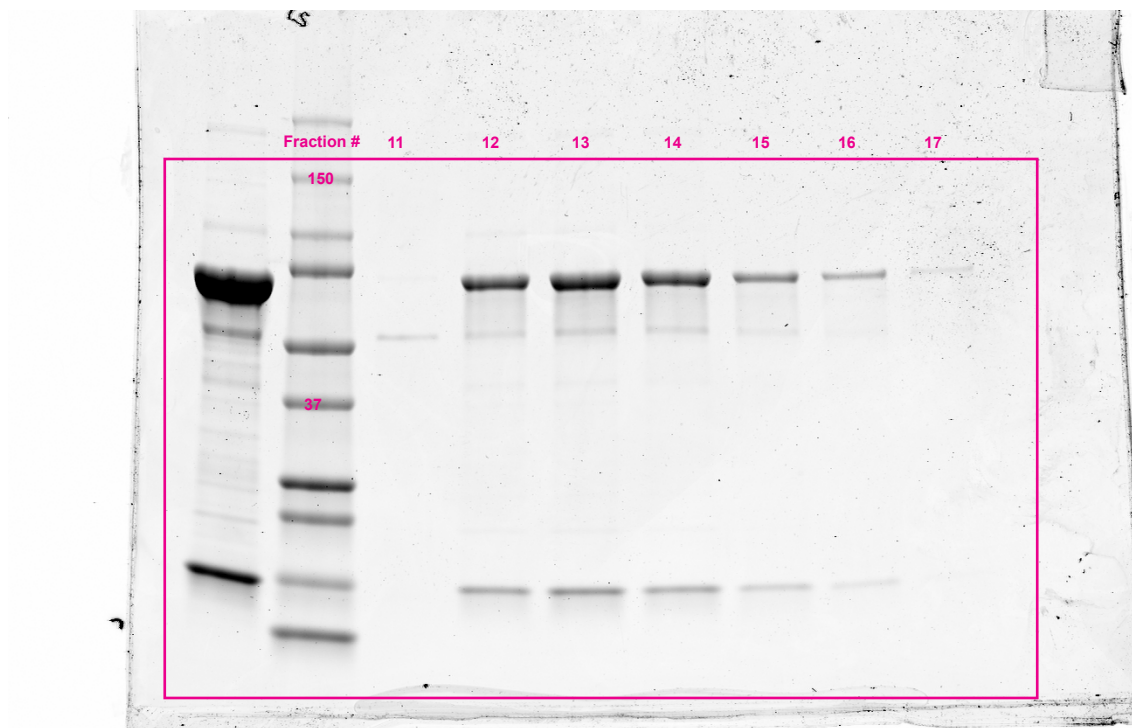

Supplement: SourceData F5 — contains original blots for Fig. 5. [file JCB_202111018_SourceDataF5.pdf]

Source Data: Figure 6A

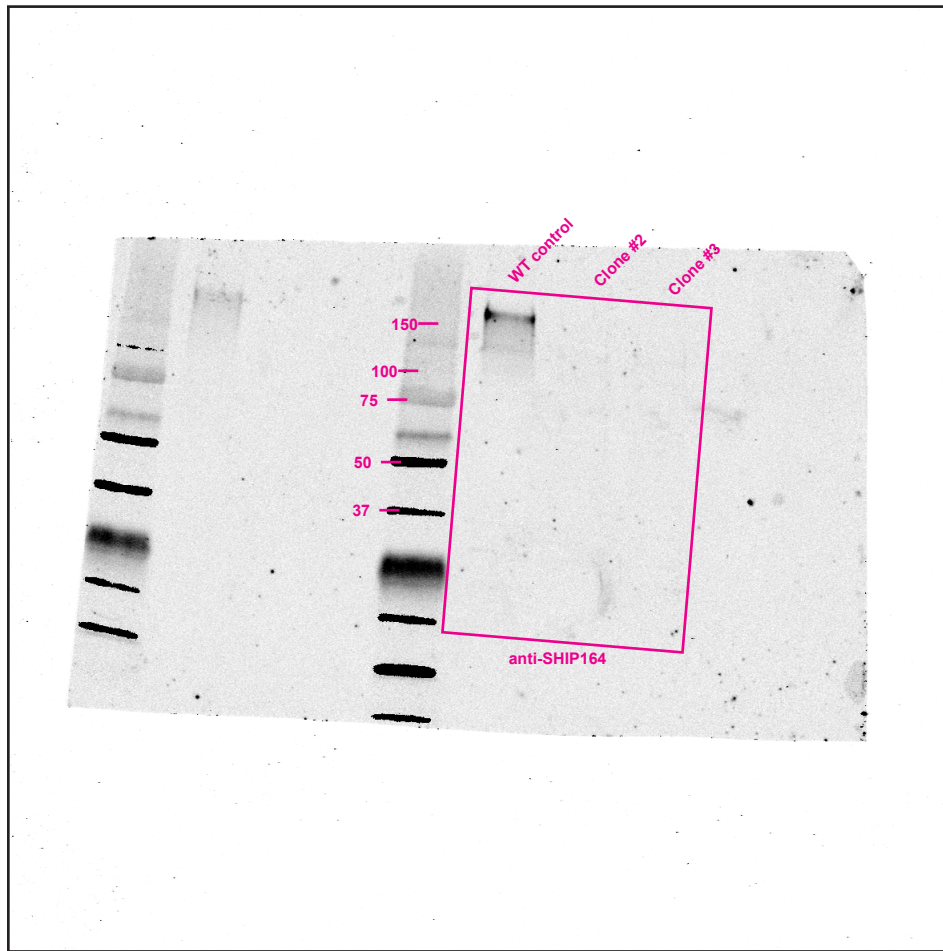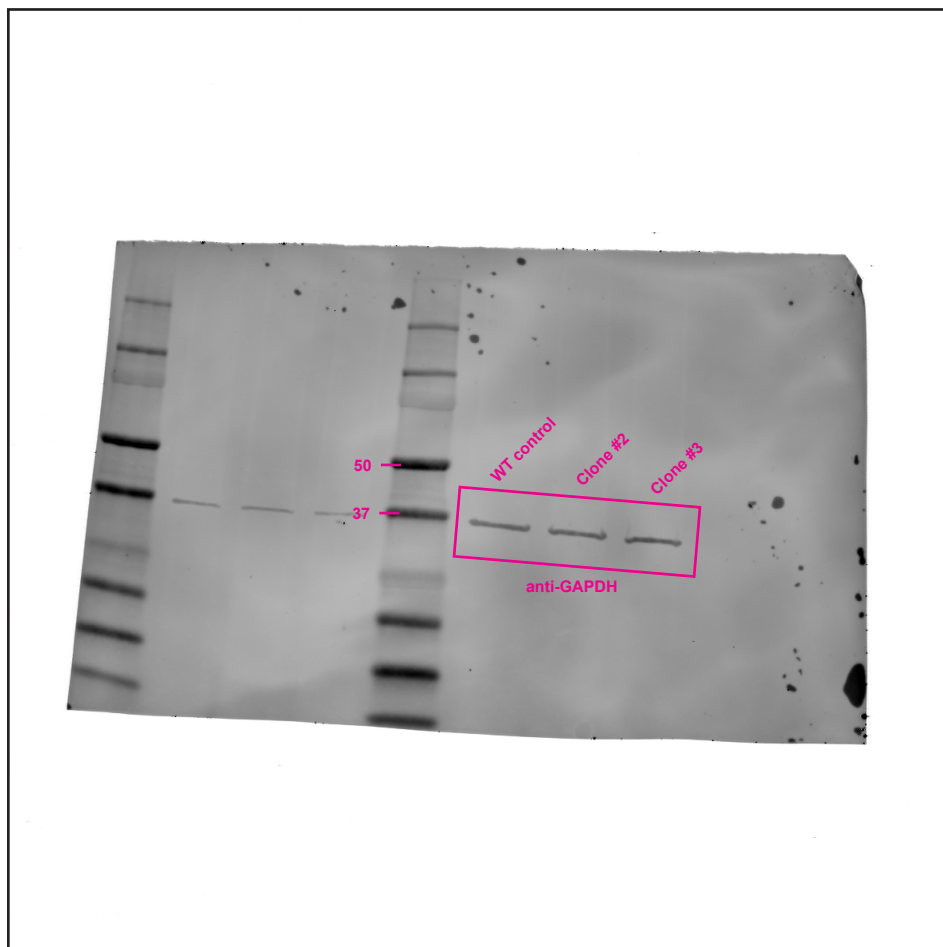

Supplement: SourceData F6 — contains original blots for Fig. 6. [file JCB_202111018_SourceDataF6.pdf]

# Source Data: Figure 7F

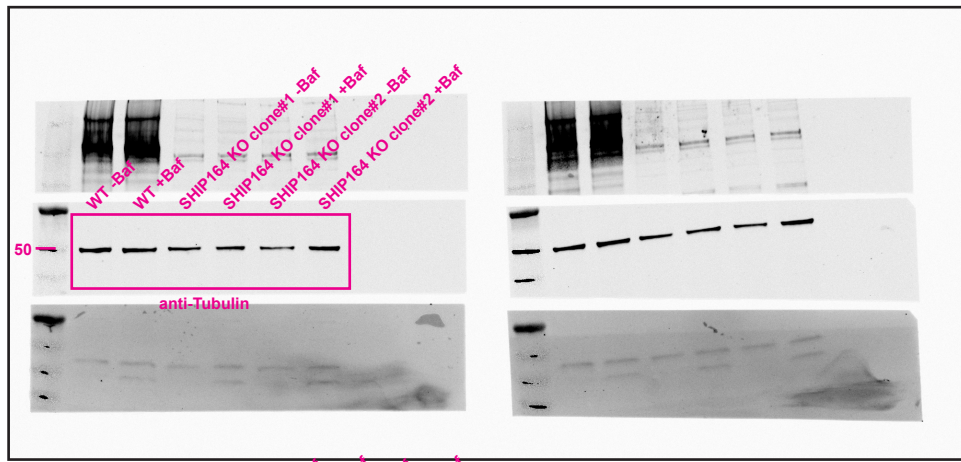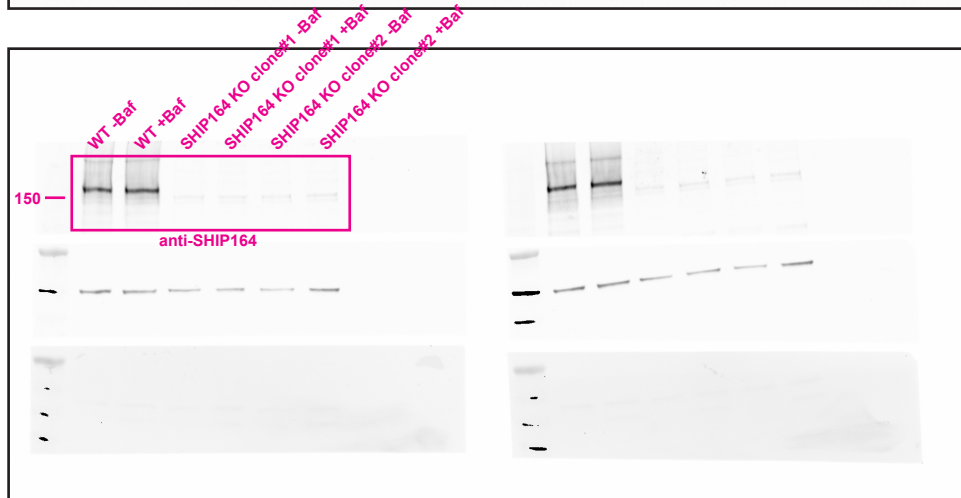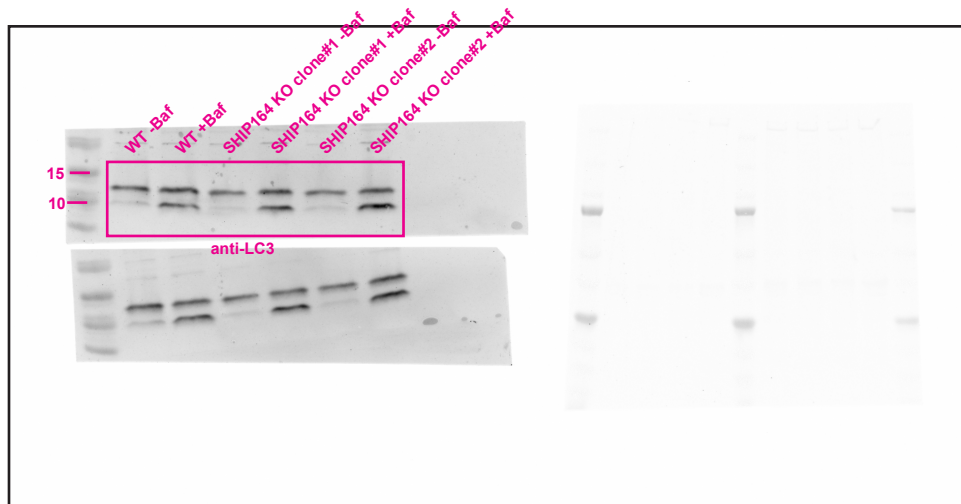

Supplement: SourceData F7 — contains original blots for Fig. 7. [file JCB_202111018_SourceDataF7.pdf]

Supplemental Fig.1 A(b)

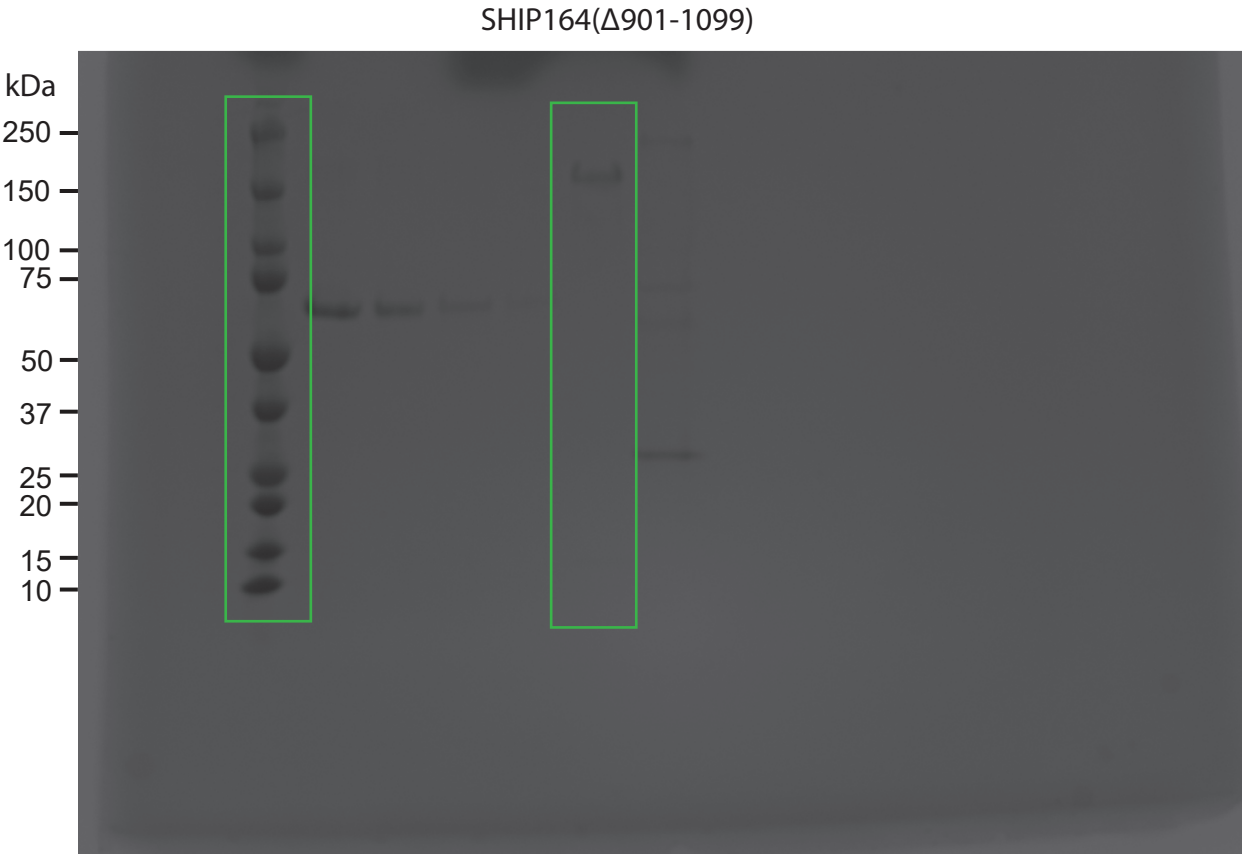

Supplement: SourceData FS1 — contains original blots for Fig. S1. [file JCB_202111018_SourceDataFS1.pdf]

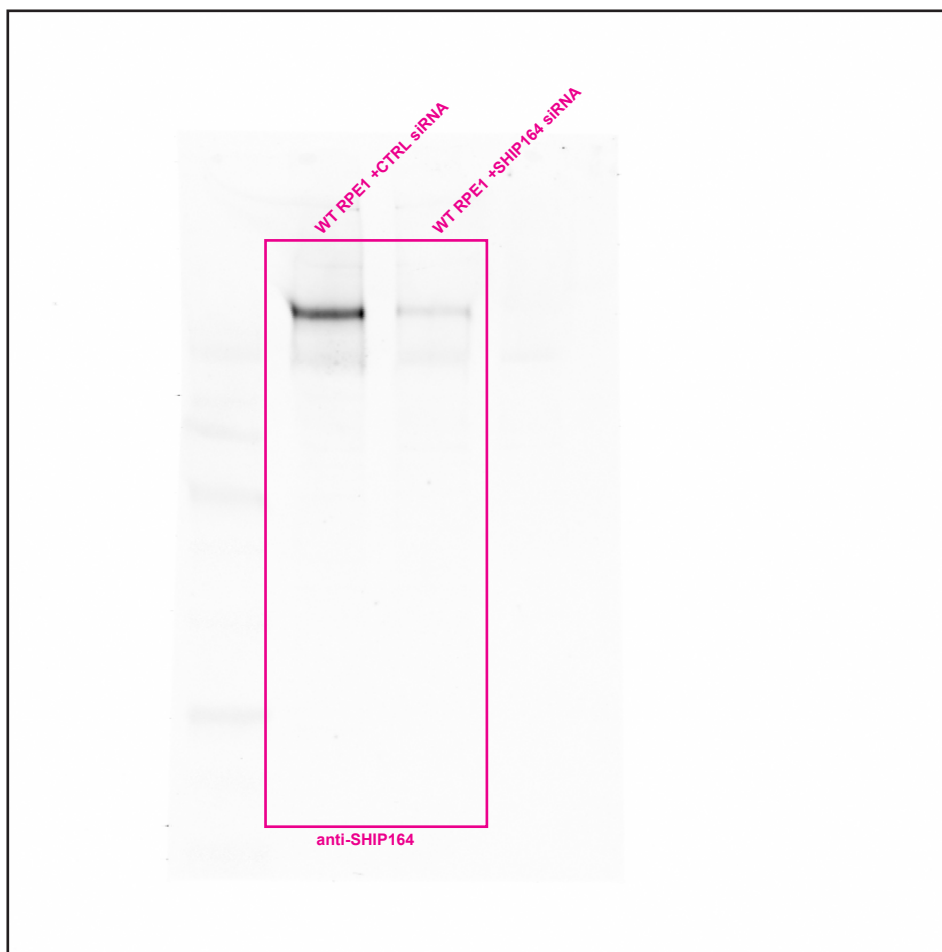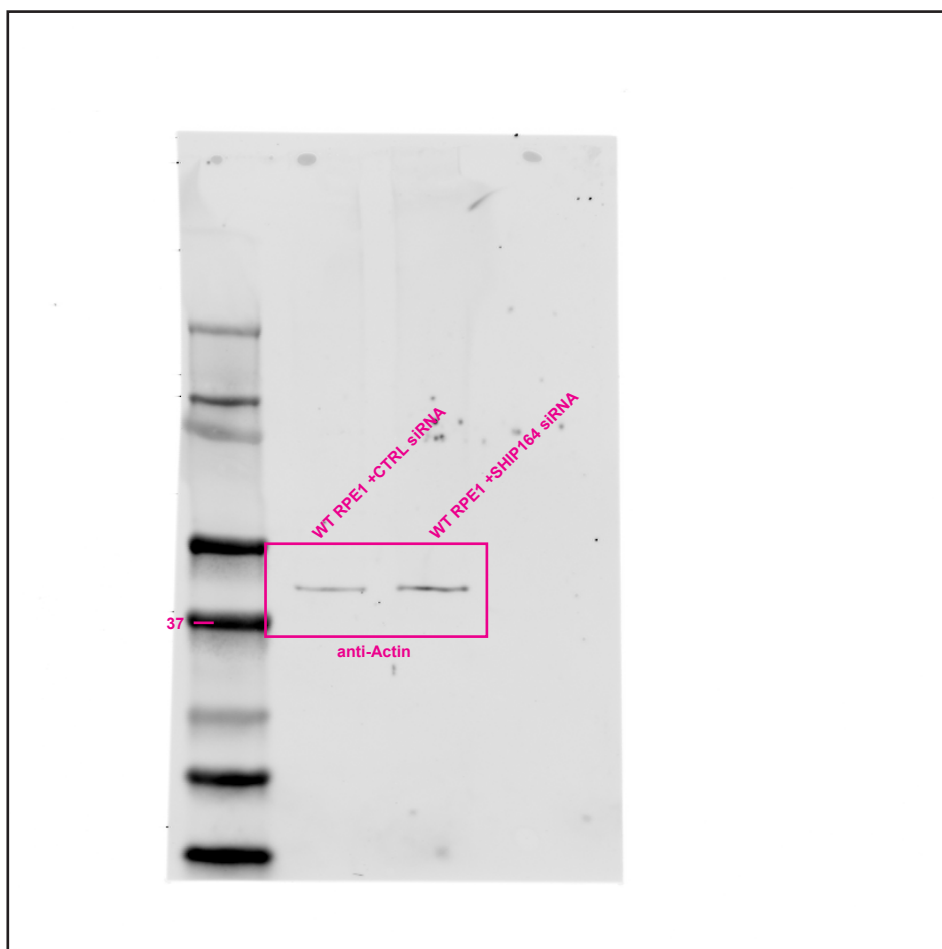

Supplement: SourceData FS4 — contains original blots for Fig. S4. [file JCB_202111018_SourceDataFS4.pdf]

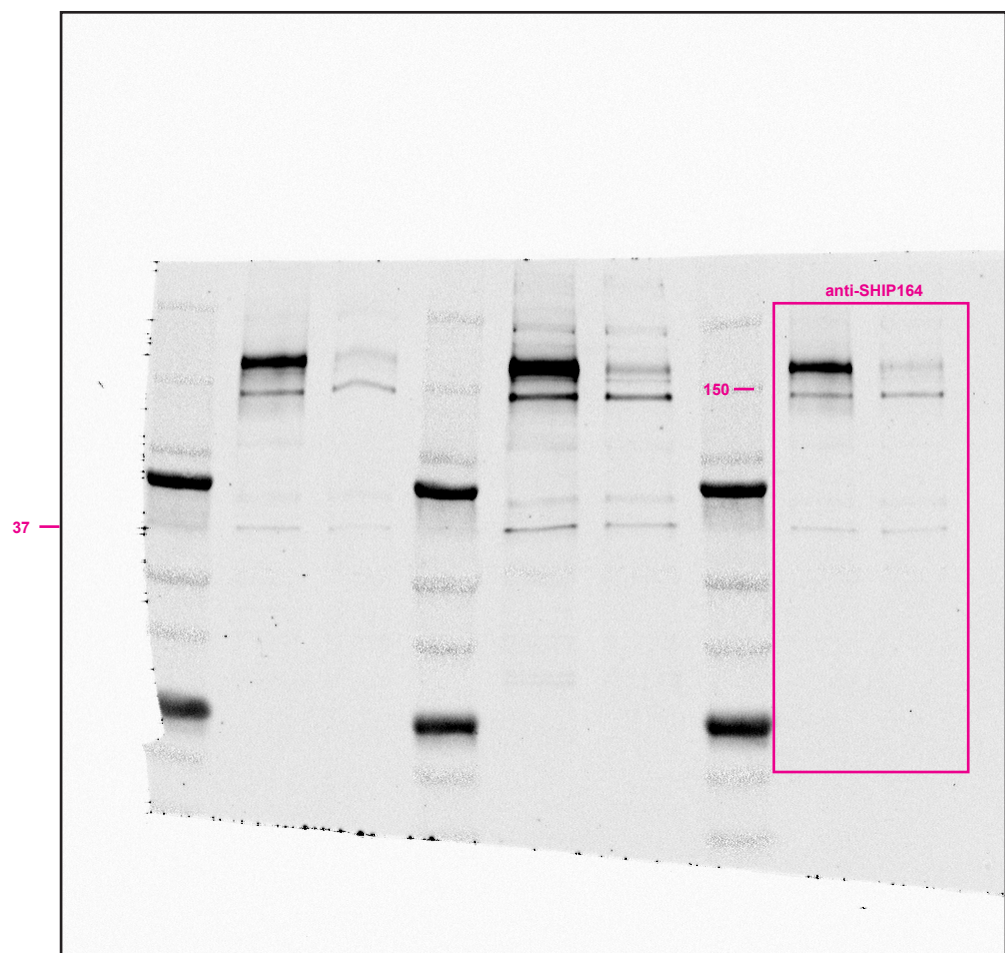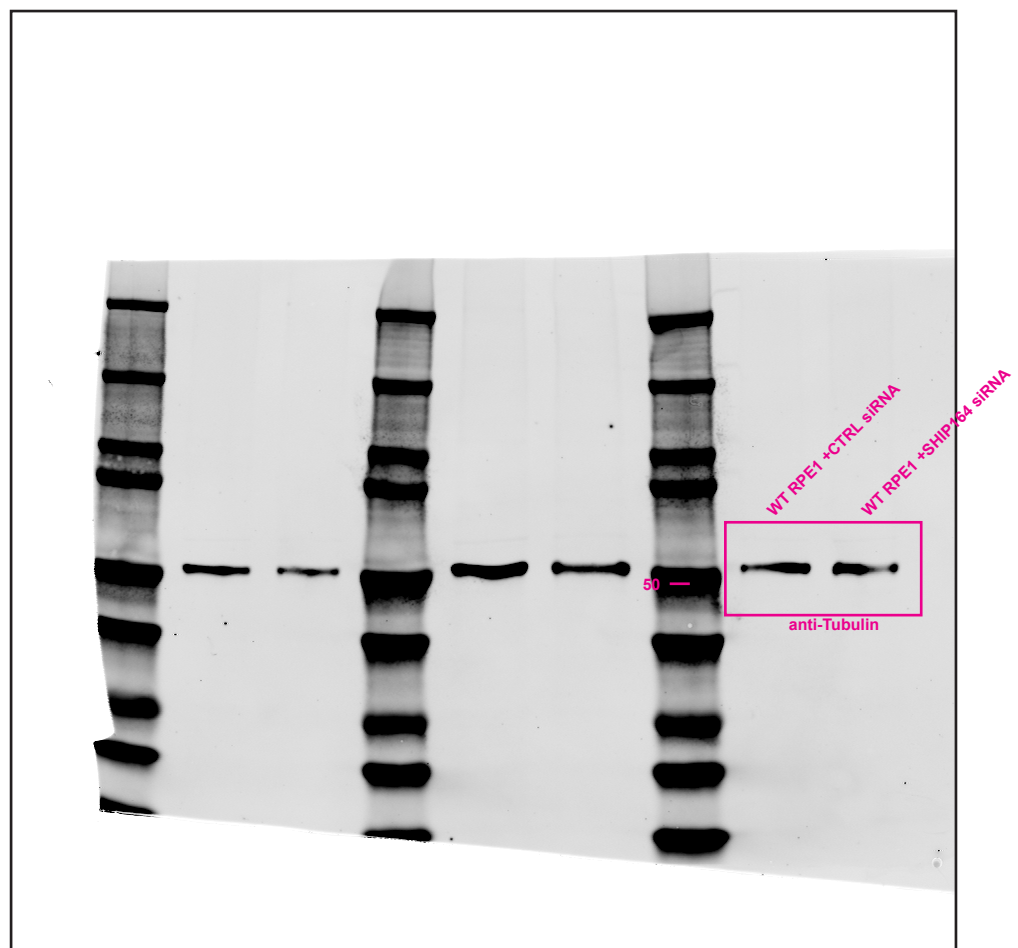

Supplement: SourceData FS5 — contains original blots for Fig. S5. [file JCB_202111018_SourceDataFS5.pdf]
